# Schirmer Tear Test Value and Corneal Lesions’ Incidence during General Anesthesia for Non-Ophthalmic Surgery in Non-Brachycephalic Dogs: A Pilot Study Comparing Three Different Lubricant Eye Drop Formulations

**DOI:** 10.3390/vetsci7010025

**Published:** 2020-02-18

**Authors:** Cristina Di Palma, Fabiana Micieli, Barbara Lamagna, Annalisa Nieddu, Valeria Uccello, Gerardo Fatone, Giancarlo Vesce

**Affiliations:** 1 Department of Veterinary Medicine and Animal Production, University of Napoli Federico II, 80137 Napoli, Italy; fabiana.micieli@unina.it (F.M.); blamagna@unina.it (B.L.); valeria.uccello@unina.it (V.U.); gerardo.fatone@unina.it (G.F.); gvesce@unina.it (G.V.); 2 Medical Manager (Veterinary Division Aurora Biofarma), 20131 Milano, Italy; anieddu@aurorabiofarma.com

**Keywords:** cornea, schirmer tear test-1, general anesthesia, lubricant eye drops, exposure keratopathy, dog

## Abstract

Aim of this blinded, prospective, randomized clinical study was to compare three different lubricant eye drops (LED) in healthy adult dogs undergoing general anaesthesia (GA) for non-ophthalmic surgery. Tear production rate was monitored by means of Schirmer tear test-1 (STT-1), and incidence of post-operative corneal abrasions/ulcerations was detected by corneal staining. A complete ophthalmic examination was performed before premedication, at extubation time and 24 h after GA in twenty-five non-brachycephalic dogs (fifty eyes) undergoing elective orthopaedic or spinal surgery procedures. Dogs were randomly allocated to one of three groups receiving as prophylactic LED either carmellose sodium (GC), or 1% hyaluronic acid (GH), or 0.25% hyaluronic acid (GL). In each eye STT-1 was repeated every hour during GA, before instilling one drop of the assigned LED. In all groups STT-1 values drastically decreased during GA, while 24 h later nine eyes (18%) had STT-1 values lower than 15 mm/minute. All of the three formulations tested were fully effective in preventing corneal ulceration (0% in all groups), while 10% of eyes reported superficial de-epithelialization. Fluorescein staining demonstrated that hourly prophylactic LED application prevented exposure keratopathy during general anesthesia in 90% of the eyes in non-brachycephalic dogs.

## 1. Introduction

Corneal abrasions are the most common ocular complication in humans during general anesthesia (GA) for non-ophthalmic surgery [1]. Adverse ocular side effects of GA are lagophthalmos, leading to exposure keratopathy (EK); loss of eyelid reflex; decreased basal tear production and reduced stability of the corneal tear film [2]. Anesthesia induced corneal lesions have been reported also in dogs [3,4,5,6,7,8], cats [9,10] and horses [11].

Eye damage under anesthesia is a preventable complication. As early as 1981 Schimidt et al. [12] reported that ocular application of a water-based 4% methylcellulose ointment could allow eye protection during GA in humans. 

In dogs, Herring et al. [4] showed that post-anesthetic Schirmer tear test (SST) values were significantly lower than baseline measurements and that an anesthesia-induced decrease of tear production persisted up to 24 h beyond the anesthetic event. These authors suggested periodic application of a lubricant eye ointment during GA as well for the following 24–36 h.

In a retrospective study on 732 dogs, Park et al. [13] reported an incidence of 1.9% of corneal ulcerations related to GA, with two cases progressing to globe-threatening complications.

In a recent prospective study [14] in dogs receiving prophylactic eye instillation of a carmellose sodium gel at induction and every 2–4 h during GA, a low incidence (0.5%) of superficial corneal ulcerations, but a high incidence of corneal epithelial erosions (18.6%) were reported.

Lubricant Eye Drops (LED) have been shown to improve corneal epithelium wound healing [15]. In a rabbit corneal epithelial scrape wound model, Zhang et al. [15] demonstrated that artificial eye drops containing 0.3% hyaluronate provided a shorter re-epithelialization time compared to drops containing 0.5% sodium carboxymethylcellulose.

The current veterinary literature lacks comparative studies about the eye protecting effects of different LEDs in dogs undergoing GA. The aim of this blinded, prospective, randomized, clinical, pilot study was to compare the effects of three different LED formulations in preventing exposure keratopathy, measuring tear production rate by means of STT-1, and scoring the incidence of post-operative corneal abrasions/ulcerations by means of corneal staining, in dogs undergoing GA for non-ophthalmic surgery.

## 2. Materials and Methods 

### 2.1. Study Design

**Figure 1:**
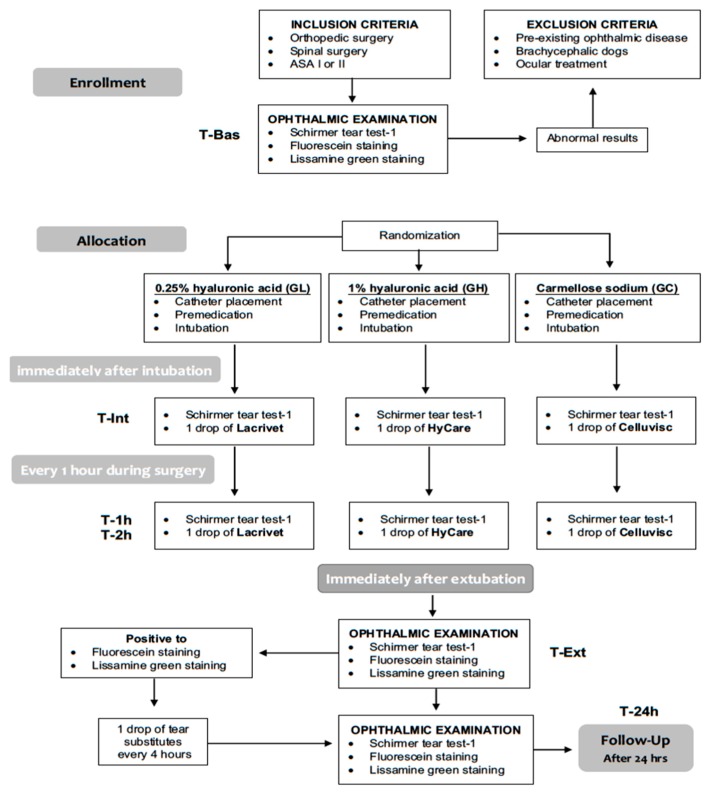
Flow chart of the study design.

This 12-month, blinded, prospective, randomized, parallel-group, pilot, clinical comparison study (Figure 1) was approved by the OPBA (Organismo Preposto al Benessere Animale) of the University of Naples “Federico II” (# 2018/0052229). The study was run from November 2018 to November 2019 at the Veterinary Teaching Hospital of the University of Napoli “Federico II”. All dogs’ owners provided written informed consent to the study.

### 2.2. Animals

Twenty-five dogs older than 6 months, scheduled to undergo elective orthopedic or spinal surgery and corresponding to physical status I or II according to the American Society of Anesthesiologists (ASA) classification, were enrolled in the study. Brachycephalic dogs and dogs with pre-existing ophthalmic disease were excluded from the study. Inclusion criteria were: baseline STT-1 values ≥15 mm/minute, absence of any ocular treatment for at least 2 months before the beginning of the study and absence of corneal abnormalities detected by fluorescein and/or lissamine-green stains. Preanesthetic evaluation included physical examination, complete blood count and serum biochemistry profile.

### 2.3. Ophthalmological Examination

The same ophthalmologist, blinded to patients’ group allocation, performed a complete ophthalmic examination (OE) on both dog’s eyes, including neuro-ophthalmological examination, STT-1 (Dina-HitexSpol, Bučovice, CZ), slit-lamp biomicroscopy (Kowa SL-15, Kowa Company Ltd., Japan), direct ophthalmoscopy without use of mydriatics (Panoptic ophthalmoscope, Welch Allyn), fluorescein staining, to evaluate the presence of corneal abrasions or ulcerations (Taurun Enterprises–Optitech Eyecare, Prayagraj, IND) and lissamine green staining, to evaluate the presence of devitalized or membrane-damaged corneal epithelial cells (Dina-HitexSpol, Bučovice, CZ). Pre-operative single eye’s STT-1 reading was recorded as baseline value (T-Bas).

### 2.4. Prophylactic Lubricant Treatment and Recording of Data

After the above OE, dogs were randomly (https://www.randomizer.org) allocated to one of three groups receiving as prophylactic LED treatment either carmellose sodium (Celluvisc^®^, 1% Dublin, IE) (GC group, n. 18 eyes), or 1% hyaluronic acid gel formulation (HyCare^®^, Chesterfield, UK) (GH group, n. 12 eyes), or 0.25% hyaluronic acid (Lacrivet^®^, Milan, Italy) (GL group, n. 20 eyes). 

STT-1 measurements were repeated immediately after endotracheal intubation (T-Int) and every hour (T-1h and T-2h) afterward, until the end of the anesthesia. After each STT-1 measurement, one drop of the LED assigned according to patient randomized group allocation, was instilled in each eye (Figure 1). A careful instillation technique was adopted and repeated each time to all eyes: a single LED drop was delivered in the eye conjunctiva avoiding eyelid contact, after occluding the tear duct by pressing the dacryocyst area.

The anesthetic protocol differed among patients at the discretion of the anesthetist, depending on individual needs. Premedication agents included acepromazine, dexmedetomidine, ketamine, midazolam and methadone or a combination of these molecules; induction agents were propofol alone, or one of the following combinations: propofol/midazolam, propofol/fentanyl, propofol/ketamine. Isoflurane was the sole GA maintenance agent for all dogs. Some of the animals received suitable preoperative loco-regional nerve blocks, either with bupivacaine or lidocaine. Perioperative analgesic treatment comprised Meloxicam (0.1 mg/Kg SC every 24 h). Prophylactic and postoperative antimicrobial treatment included cefazolin (20 mg/Kg IV). All patients were hospitalized for at least 24 h postop.

The same ophthalmologist, blind to the individually assigned LED treatment, performed all OE including STT-1, slit-lamp biomicroscopy, fluorescein and lissamine green staining, within five minutes following extubation (T-Ext) and again 24 h after anesthetic recovery (T-24h). In case of corneal lesions, the same LED used for prophylactic eye protection was applied every 4 h for the following 24 h. In case of corneal lesions persisting beyond T-24, the same treatment was continued until the corneal lesion resolved. Size and location of the corneal lesions were recorded for each eye. The corneal surface was ideally divided into three horizontal segments as described by Wan T. et al. [16]; for each of those segments, the position (medial central or lateral), the extent (whole or partial) and the depth (abrasion or ulceration) of the corneal lesion were recorded. 

### 2.5. Statistical Analysis

All numerical data were recorded using a computerized spread-sheet (Microsoft Excel 2011) and imported into a program for statistical analysis (SPSS, IBM Armonk, NY, USA). All data were checked for normality of distribution using a Shapiro–Wilk test. Results are expressed as means ± standard deviation (SD) when normally distributed, whereas non-parametric data are reported as the median (min, max).

Differences in presence of corneal epithelial lesions between groups were compared using Chi-squared test. Differences in STT-1 values between eyes (right (R) vs. left (L)) were analysed using Wilcoxon signed-rank. In absence of statistically significant difference between subject’s R and L eye, their median STT-1 value was used for the further analysis. Data were examined using Mann–Whitney U-test for comparisons between groups and Turkey test for point-to-point comparisons among the different times in each group. Values of *p* < 0.05 were considered significant in all analyses.

## 3. Results

**Figure 2:**
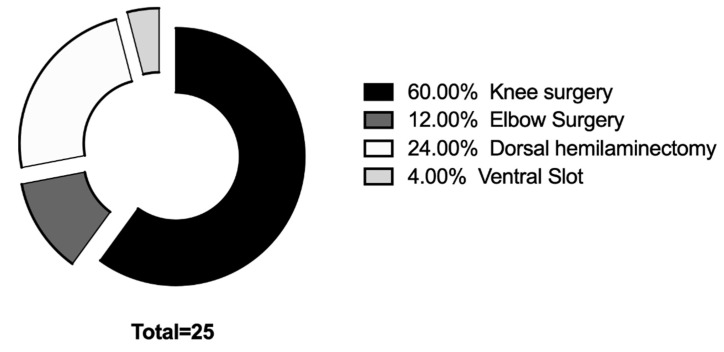
Distribution of surgical procedures included in the study.

Twenty-five dogs (fifty eyes) were included in the study. The average age was (Mean ± SD) 59.16 ± 28.09 months; the median weight was 10.55 kg (4–45 kg). There were 16 males and nine females. Median anesthesia time was 137 min (range 75–242 min). Distribution of surgical procedures was reported in Figure 2. No statistically significant differences were found among groups. for the duration of anesthesia (*p* = 0.134). 

Median and range values of STT-1 at different times are reported in Table 1 for each group. Statistically significant differences (P) in STT-1 values were found among groups at T-1h (*p* = 0.018) and at T-24h (*p* = 0.016). At T-Ind STT-1 values decreased in all groups from T-Bas without significant differences among groups (*p* = 0.066). Dogs in GL had significantly higher STT-1 values compared to dogs in groups GC at T-1h (*p* = 0.023). Furthermore, STT-1 values were higher in GL compared to GC and GH at T-24h (*p* = 0.030 and *p* = 0.021 respectively). No statistically significant differences were found between groups in the interval between last LED instillation and T-Ext (*p* = 0.256).

Fluorescein stain uptake was observed in 10% of eyes at T-Ext (10% in GL, 11% in GC and 8% in GH) and in 6% of the eyes at T-24h (10% in GL, 5% in GC and 0% in GH). In all cases a corneal abrasion was recorded. No statistically significant differences in fluorescein stain uptake were found between groups at T-Ext and at T-24h. Lissamine green uptake was evident in 4% of eyes at T-Ext (8% in GH, 5% in GL and 0% in GC) No statistically significant differences were found between groups. None of the eyes reported corneal ulcerations. Four out of five corneal abrasions observed at T-Ext, resolved within the first 24 h; only in one case (GC) a corneal abrasion persisted behind T-24h. Among the five corneal lesions detected by fluorescein stain uptake at T-Ext, one was a central linear lesion in the central segment and four were point-like lesions localized in the lateral part of the cornea, three in the superior segment and one in the central segment. The three lesions detected at T-24h were all localized in the lateral part of the superior segment. Lissamine green stained two corneal lesions at T-Ext, one on the lateral part of the superior segment and one on the lateral part of the central segment.

In our study, none of the dogs reporting corneal abrasions showed clinical symptoms (i.e., blinking, blepharospasm, ocular pain and/or epiphora) related to EK. All three formulations tested in our study were equally effective favoring corneal healing.

## 4. Discussion

The results of our study confirmed that during and after GA tear production rate is decreased in dogs.

Nonetheless, even if no LED is able to increase tear production rate, their use is recommended to reduce corneal dehydration rate and to increase corneal wettability [17].

In the present study, prophylactic application of a 0.25% hyaluronic acid LED (GL) produced significantly higher STT-1 values compared to a carmellose sodium based LED (GC) at T-1h (median values 3.75 mm/minute; 0 mm/minute respectively) and at T-24h (median values 18.5 mm/minute; 14.5 mm/minute).

In human patients with moderately dry eyes and with superficial keratitis, sodium hyaluronate has been shown to promote faster recovery from keratitis compared to carboxymethylcellulose treatment [18]. In those patients, an improvement of corneal surface regularity index and an earlier healing of the cornea were ascribed to a hyaluronate protective coating effect, preventing progression of the keratitis [18]. Sodium hyaluronate positive effects were endorsed to its enhanced pseudoplastic and elastic properties, comparable to those of human natural tears, contributing to the maintenance of a stable tear film [19]. 

Despite the positive effect of sodium hyaluronate on tear film stability, the present pilot study showed that of the two hyaluronic acid (HA) treated groups, the one receiving a lesser concentration (GL = 0.25%) showed higher SST-1 readings compared to the group receiving a more concentrated HA solution (GC = 1%). 

In our study, 24 h after GA, nine eyes (18%) had values lower than 15 mm/min. Such prolonged inhibitory effect of GA on tear production rate, has been disputed in dogs undergoing inhalant anesthesia by Shepard MK et Coll. [5], reporting that aqueous tear production returned to baseline values immediately after recovery and kept so until 10 h after isoflurane and desflurane anesthesia.

In agreement with our findings, Herring et al. reported significant decreases in STT-1 values 24 h after GA in dogs missing any LED treatment [4], with a mean value of 15.8 mm per minute (range, 4–18 mm per minute). Similarly, in the study of Dawson et al., STT-1 readings remained significantly lower 24 h post-GA when compared to baseline measurements [14]. 

The overall incidence of corneal abrasions found in in the present study (10% of eyes at T-Ext and 6% of eyes at T-24h) is moderately lower than that reported by Dawson et al. [14] (18.6% of eyes after 24 h) in dogs undergoing GA under the protection of a carmellose sodium based gel. In the latter study, however, also brachycephalic breeds and dogs with baseline values of STT <15 mm/min were included, while eye drops were instilled every 2–4 h. Such disparities could account for our more favorable findings, including the absence of corneal ulcerations, whose incidence reached 0.5% in Dawson’s study. 

In our study, no corneal ulcers were diagnosed, while the incidence of corneal abrasions at T-Ext was 10% (GL) and 8% (GH) in dogs treated by prophylactic application of two hyaluronic acid LED formulations and 11% in dogs treated by the carmellose sodium LED. Our results demonstrate that all of the three formulations tested in healthy dogs’ eyes, when administered hourly during GA, are effective in preventing corneal ulceration and in reducing the incidence of corneal abrasions. 

This study has several limitations. The small number of dogs tested is a main limitation. Also, our results could have been supported by performing conjunctival impression cytology, conjunctival swabs and by measuring tear’s break up time. Further limitations are the lack of a standardized anesthesia protocol and the varied surgical procedures. 

New studies are needed to compare the effects of different eye drops formulations in healthy dogs, as well in brachycephalic breeds and in patients bearing ocular diseases undergoing anesthesia and surgery for non-ophthalmic diseases. Furthermore, to the best of the author’s knowledge, there are no data about the incidence of corneal lesions in dogs undergoing GA without eye protection. In human settings, Grover et al. [20] showed that 90% of eyes enduring post-anesthetic corneal epithelial defects were untreated, compared to 3.3% in eye ointment treated patients.

The overall finding of corneal abrasions in dogs undergoing GA under the protection of a topical LED shows that, like in humans, such widespread practice, lacking standardized protocols, can be ineffective against EK in animals as well. Additional effective measures adopted in human anesthesia and in intensive care units for preventing EK, reviewed by Rosenberg JB et al. [21], include taping of the eyelids, covering the eyes with moistened gauze, use of ophthalmic ointments and of eye moisture chambers. 

## 5. Conclusions 

The present study compares the incidence of perioperative corneal lesions following instillation of three different LEDs in non-brachycephalic dogs undergoing elective orthopaedic or spinal procedures. Prophylactic application of one drop of any of the three scrutinized LEDs every hour provided 100% protection against corneal ulceration and 90% protection against corneal abrasions in dogs undergoing GA. The three formulations tested were comparable in successfully preventing corneal lesions.

Perioperative prevention of corneal lesions can be effectively achieved by several means, such as taping of the eyelids, applying eye ointments or LEDs. None of these measures promotes tear production or guarantees absolute avoidance of exposure keratopathy. AAHA Anesthesia Guidelines for Dogs and Cats endorse applying a corneal lubricant after induction and repeating it throughout anesthesia and recovery, to decrease incidence and severity of corneal damage [22]. It is the responsibility of the anesthetist to protect the patient’s eyes and to adopt prophylactic exposure keratopathy measures, paying constant attention to patient’s head and eyes. Hourly application of a LED during and after general anesthesia, as well postoperative slit lamp ophthalmic examination should be judiciously implemented in veterinary patients, even in absence of specific, standardized eye protection protocols.

## Figures and Tables

**Table 1:** Median and range values of STT-1 at different times in three groups of dogs undergone GA, treated by prophylactic application of three different lubricant eye drops (LED).

Items	STT-1 (mm/min)			
Time Point	GL	GC	GH	*p*-Value
T-Bas	22.7 (16.5; 29)	18.5 (15; 22.5)	18.5 (17.5; 20.5)	0.066
T-Int	9 (3.6; 16.5)	7.3 (1.5; 14)	8.2 (3.5; 17)	0.567
T-1h	3.75 (0; 6.5)	0 (0; 3.5) ^a^	1.7 (0.5; 7)	0.018 *
T-2h	5 (0; 7.5)	0.2 (0; 2.5)	1 (1; 5)	0.078
T-Ext	4.7 (2; 9.5)	0.5 (0; 10)	3 (0.5; 5.5)	0.290
T-24h	18.5 (16.5; 25)	14.5 (11.5; 22) ^a^	15 (10; 18) ^a^	0.016 *

STT-1: Schirmer tear test-1, T-Bas: Baseline, T-Int: immediately after intubation, T-1h: 1 h after intubation, T-2h: 2 h after intubation, T-Ext: after extubation, T-24h: 24 h after anesthetic recovery, GL: group treated by 0.25% hyaluronic acid (Lacrivet^®^), GC: group treated by carmellose sodium (Celluvisc^®^ 1%), GH: group treated by 1% hyaluronic acid (HyCare^®^). * Significant difference between groups. ^a^ Significant different from GL.
